# Behavioral Characterization of the Effects of Cannabis Smoke and Anandamide in Rats

**DOI:** 10.1371/journal.pone.0153327

**Published:** 2016-04-11

**Authors:** Adriaan W. Bruijnzeel, Xiaoli Qi, Lidia V. Guzhva, Shannon Wall, Jie V. Deng, Mark S. Gold, Marcelo Febo, Barry Setlow

**Affiliations:** 1 Department of Psychiatry, University of Florida, Gainesville, Florida, United States of America; 2 Department of Neuroscience, University of Florida, Gainesville, Florida, United States of America; 3 Center for Addiction Research and Education, University of Florida, Gainesville, Florida, United States of America; The University of Texas at Austin, UNITED STATES

## Abstract

Cannabis is the most widely used illicit drug in the world. Delta-9-tetrahydrocannabinol (Δ9-THC) is the main psychoactive component of cannabis and its effects have been well-studied. However, cannabis contains many other cannabinoids that affect brain function. Therefore, these studies investigated the effect of cannabis smoke exposure on locomotor activity, rearing, anxiety-like behavior, and the development of dependence in rats. It was also investigated if cannabis smoke exposure leads to tolerance to the locomotor-suppressant effects of the endogenous cannabinoid anandamide. Cannabis smoke was generated by burning 5.7% Δ9-THC cannabis cigarettes in a smoking machine. The effect of cannabis smoke on the behavior of rats in a small and large open field and an elevated plus maze was evaluated. Cannabis smoke exposure induced a brief increase in locomotor activity followed by a prolonged decrease in locomotor activity and rearing in the 30-min small open field test. The cannabinoid receptor type 1 (CB_1_) receptor antagonist rimonabant increased locomotor activity and prevented the smoke-induced decrease in rearing. Smoke exposure also increased locomotor activity in the 5-min large open field test and the elevated plus maze test. The smoke exposed rats spent more time in the center zone of the large open field, which is indicative of a decrease in anxiety-like behavior. A high dose of anandamide decreased locomotor activity and rearing in the small open field and this was not prevented by rimonabant or pre-exposure to cannabis smoke. Serum Δ9-THC levels were 225 ng/ml after smoke exposure, which is similar to levels in humans after smoking cannabis. Exposure to cannabis smoke led to dependence as indicated by more rimonabant-precipitated somatic withdrawal signs in the cannabis smoke exposed rats than in the air-control rats. In conclusion, chronic cannabis smoke exposure in rats leads to clinically relevant Δ9-THC levels, dependence, and has a biphasic effect on locomotor activity.

## Introduction

Cannabis is the most widely used illicit drug in the world. The United Nations Office on Drugs and Crime estimates that 2.7–4.9% of adults worldwide use cannabis [1]. The prevalence of cannabis use is particularly high in Ghana (21.5%), Zambia (17.7%), Canada (17.0%), the United States of America (12.3%), and New Zealand (13.3%)[2]. The subjective effects of cannabis and its main psychoactive component, delta-9-tetrahydrocannabinol (Δ9-THC), include relaxation, mild euphoria, perceptual changes, intense laughter, and talkativeness [3, 4]. However, cannabis use can also have adverse effects including impaired memory function and paranoia [5, 6]. Chronic cannabis use may lead to dependence [7, 8], and cessation of chronic use can lead to affective withdrawal symptoms including increased anxiety, irritability, aggression, intense craving for cannabis, difficulty sleeping, and somatic complaints [9–11]. There are currently no pharmacological treatments for cannabis addiction.

In addition to Δ9-THC, several other cannabinoids have been isolated from cannabis that can affect brain function. More than 80 cannabinoids have been identified and they can be divided into 10 different classes based on their chemical structure (e.g., cannabidiol, cannabinol, Δ9-THC, etc.)[12, 13]. Plant cannabinoids and endogenous cannabinoids mediate their effects via the activation of two cannabinoid receptors, namely the cannabinoid receptor type 1 (CB_1_) and receptor type 2 (CB_2_) [14, 15]. Both receptors are coupled to G_i_/G_o_ proteins and stimulation of these receptors decreases cAMP levels [16]. The highly selective CB_1_ receptor antagonist/partial agonist rimonabant (SR 141716A) has been shown to block most of the psychoactive effects of Δ9-THC as well as Δ9-THC self-administration [17–19]. High levels of CB_1_ receptors have been detected in the basal ganglia (caudate putamen, globus pallidus, and substantia nigra), molecular layer of the cerebral cortex, and subregions of the hippocampus including the CA3 region and the dentate gyrus [20, 21]. The localization of these receptors points to a critical role for the cannabinoid system in cognition and motor function. Relatively low levels of CB_1_ receptors have been detected in brain areas that play a role in reward signaling such as the ventral tegmental area and nucleus accumbens shell [21, 22].

Animal studies have shown that high doses of ∆9-THC [23] and the endogenous cannabinoid anandamide [24] decrease locomotor activity, and that chronic Δ9-THC administration leads to the development of dependence [25–27]. However, cannabis contains many different cannabinoids and the combined effect of these cannabinoids on the brain is poorly understood. It has been suggested that other cannabinoids in cannabis may have additive, synergistic, or opposing effects with respect to those of Δ9-THC [28]. Furthermore, inhalation is the main route of cannabis self-administration in humans whereas in animal studies cannabinoids are usually injected [29]. To mimic human cannabis smoke exposure, we developed an animal model in which freely moving rats were exposed to cannabis smoke from 5.7% Δ9-THC cigarettes [30]. Chronic cannabis use can lead to depression and the endogenous cannabinoid anandamide improves mood states [31, 32]. Therefore, it was investigated whether cannabis smoke exposure decreases the sensitivity to anandamide. The goal of the studies using this model was three-fold. 1) Determine the effects of cannabis smoke on exploratory behavior, development of dependence, and serum Δ9-THC levels. 2) Determine the acute effects of anandamide on exploratory behavior. 3) Determine if chronic cannabis smoke exposure leads to tolerance to the acute behavioral effects of anandamide.

## Material and Methods

### Subjects

Male Wistar rats (200–225 g upon arrival, Charles River, Raleigh, NC) were used for all experiments.

When the rats arrived in the vivarium they were about 50 days of age and the experiments started at least one week later. Therefore, the rats had reached adulthood when the smoke exposure sessions started [33]. The animals were housed (2 per cage) in a temperature and humidity-controlled vivarium and maintained on a 12-h reversed light-dark cycle (lights off at 8 AM), with free access to food and water at all times. All experiments were conducted during the dark phase. All subjects were treated in accordance with National Institutes of Health guidelines regarding the principles of animal care. Animal facilities and experimental protocols were in accordance with the Association for the Assessment and Accreditation of Laboratory Animal Care (AAALAC) and approved by the University of Florida Institutional Animal Care and Use Committee.

### Drugs

Rimonabant hydrochloride (SR 141716A) and anandamide (in Tocrisolve^TM^ 100, 1:4 ratio of soya oil/water and emulsified with the block co-polymer Pluronic F68) were purchased from Tocris Bioscience (Bristol, United Kingdom). Tocrisolve^TM^ 100 was used as vehicle in the anandamide studies (Tocris Bioscience, Bristol, United Kingdom). Rimonabant was dissolved in a vehicle of Tween 80 (5% volume/volume, v/v), DMSO (20% v/v), and sterile saline (75% v/v). The volume percentages refer to the final rimonabant solution. Both rimonabant and anandamide were administered intraperitoneally (ip) at a volume of 1 ml/kg. Cannabis cigarettes (5.7% Δ9-THC, 0.02% cannabidiol) were kindly provided by the NIDA Drug Supply Program. The cannabis cigarettes were stored at -20°C. About 24 h before being used, the cigarettes were removed from the freezer and placed in an airtight humidity chamber with a small dish (10 x 10 cm) containing 0.5 cm of water (95% humidity) and kept at room temperature (22–24°C). The cigarettes were used within one hour of being removed from the humidity chamber.

### Experimental design

#### Experiment 1. Effect of cannabis smoke on exploratory behavior and development of dependence

**Fig 1:**
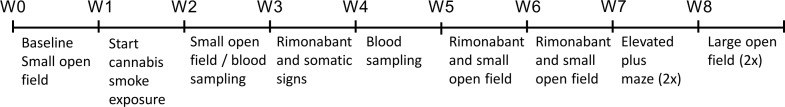
Timeline of experimental procedures in experiment 1. Abbreviation: W, week.

For this experiment 20 rats were used (10 air-control rats and 10 cannabis rats, see Fig 1 for a timeline of the tests conducted in this experiment). Prior to the onset of the experiment, the rats were handled for 3 days and their body weights were recorded. Five days before the onset of the smoke exposure sessions (day -5) the rats were tested in the small open field (30 min) to assess baseline activity levels. The cannabis smoke exposure sessions (1 h per day, 5 days per week) started on day 1 and continued for 8 weeks. To investigate the acute effects of cannabis smoke exposure on the behavior of rats (locomotor activity and rearing), they were placed in the small open field (30 min) immediately after smoke exposure on days 10 and 11 (calendar days, week 2). Half the animals were tested on day 10 and the other half on day 11. Blood samples were collected immediately after the smoke exposure session on days 12 (week 2) and 26 (week 4). To investigate if exposure to cannabis smoke leads to the development of dependence, rimonabant-precipitated (5 mg/kg, ip) somatic withdrawal signs were evaluated 4 h after smoke exposure on days 16 and 19 (week 3). Rimonabant was administered 10 min before testing. The effect of rimonabant (5 mg/kg, ip) on exploration of the small open field (30 min pre-test, and 45 min post drug test) was investigated on days 31, 32, 38, and 39 (weeks 5 and 6). On day 42 (week 7), the animals were placed in the elevated plus maze (for 5 min) 48 h after the previous smoke exposure, and on the following day (day 43) the animals were placed on the same apparatus (for 5 min) immediately after smoke exposure. On day 49 (week 8), the animals were placed in the large open field (for 5 min), 48 h after the previous smoke exposure, and on the following day (day 50) the animals were placed in the same apparatus (for 5 min) immediately after smoke exposure.

#### Experiment 2. Effect of anandamide on exploratory behavior

For this experiment 50 rats were used, and the effects of 5 doses of anandamide (0, 0.01, 0.1, 1, 10 mg/kg) on exploration of the small and large open field was investigated. Before the tests, the animals were handled for 3 days and their body weights were recorded. Then the effect of anandamide on exploration of the small open field was investigated. After a wash-out period of fourteen days the effect of anandamide on exploration of the large open field was investigated. The rats were placed in the small (45 min) or large (10 min) open field immediately after they received anandamide. The dose received by each rat for the large open field test was counterbalanced with respect to the dose received in the small open field test. Two weeks after the large open field test, it was investigated if rimonabant (5 mg/kg, ip) diminishes the effects of a high dose of anandamide (10 mg/kg, ip) on the behavior of rats in the small open field (15 min). Rimonabant was administered 10 min before anandamide.

#### Experiment 3. Effect of cannabis smoke on anandamide-induced changes in exploratory behavior

For this experiment 40 rats were used. The goal of this experiment was to determine if chronic exposure to cannabis smoke (2 weeks, 5 days per week) leads to tolerance to the effects of anandamide (10 mg/kg) on locomotor activity. This experiment consisted of the following groups: air-vehicle (n = 10), air-anandamide (n = 10), cannabis-vehicle (n = 10), cannabis-anandamide (n = 10). Anandamide or vehicle injections and testing in the small open field (45 min) took place 24 h after the final exposure session. The rats were placed in the small open field immediately after they received anandamide.

### Cannabis smoke exposure

Freely moving rats were exposed to cannabis smoke using an apparatus similar to that used previously in our laboratory to expose rats to tobacco smoke [34–37]. The rats were exposed to cannabis smoke in standard polycarbonate rodent cages (38 x 28 x 20 cm; L x W x H) with corncob bedding and wire tops. The rats were not restrained (whole body exposure) during the cannabis smoke exposure sessions and water was freely available. The rats were moved to the exposure cages immediately before the smoke exposure sessions and returned to their home cages after the exposure sessions. Cannabis smoke was generated using a microprocessor-controlled cigarette smoking-machine (model TE-10, Teague Enterprises, Davis, CA)[30]. Smoke was generated by burning cannabis cigarettes using a standardized smoking procedure (35 cm^3^ puff volume, 1 puff per minute, 2 seconds per puff). Mainstream and sidestream smoke was transported to a mixing and diluting chamber. The smoke was diluted with air to a concentration of about 550 mg of total suspended particles (TSP) per m^3^ before being introduced into the exposure chambers. Exposure conditions were monitored for carbon monoxide (CO) and TSP levels. CO levels were assessed using a continuous CO analyzer that accurately measures CO levels between 0 and 2000 parts per million (Monoxor III, Bacharach, New Kensington, PA USA). In order to measure TSP levels, smoke was pumped out of the chamber through a pre-weighed filter (Pallflex Emfab Filter, Pall Corporation, Port Washington, NY USA) for 5 min. The total suspended particulate matter per cubic meter was calculated by dividing the weight increase of the filter by the volume of the airflow through the filter. The rats in the cannabis group were exposed to smoke for 1 h per day. During this 1-h period 5 cannabis cigarettes were burned (10 min per cigarette with a 2 min break between cigarettes). In experiment 1, the average TSP level was 614 ± 31 mg/m^3^ and the CO level was 285 ± 15 ppm. In experiment 3, the TSP level was 569 ± 53 mg/m^3^ and the CO level was 200 ± 16 ppm.

### Blood sampling and Δ9-THC ELISA

Rats were lightly anesthetized with isoflurane and blood samples (500 μl) were collected by puncturing the lateral saphenous vein with a 20G needle. Blood was collected in microcentrifuge tubes and left at room temperature for 3 h. The samples were centrifuged at 3,000 x g for 10 min at 4°C and then serum was collected. The samples were stored in a -80°C freezer until later use. Delta9-THC levels were determined using an ELISA kit (Catalog #. 5013, Bioo Scientific, Austin, TX, USA) according to the manufacturer’s instructions.

### Small open field test

The small open field test was conducted as described previously [38]. The number of horizontal and vertical beam breaks was measured with an automated animal activity cage system (VersaMax Animal Activity Monitoring System, AccuScan Instruments, Columbus, OH, USA). Horizontal beam breaks reflect locomotor activity and vertical beam breaks reflect rearing. The system consisted of four animal activity cages made of clear acrylic (40 cm × 40 cm × 30 cm; length [L] x width [W] x height [H]), with 16 equally spaced (2.5 cm) infrared beams across the length and width of the cage at a height of 2 cm from the cage floor (horizontal activity beams). An additional set of 16 infrared beams was located at a height of 14 cm from the cage floor (vertical activity beams). All beams were connected to a VersaMax analyzer which sent information to a computer that displayed beam data through Windows-based software (VersaDat software). The small open field test was conducted in a darkened room, and the cages were cleaned with a Nolvasan solution (chlorhexidine diacetate) between animals.

### Large open field test

The large open field test was conducted in a dimly lit room (75 lux). The open field consisted of a large square arena measuring 120 x 120 x 60 cm (L x W x H). The arena was made of black high-density polyethylene panels that were screwed together and placed on a plastic bottom plate (Faulkner Plastics, Miami, FL). The behavior of the animals was recorded with a camera mounted above the arena and analyzed with EthoVision XT 8.5 software (Noldus Information Technology, Leesburg, VA). The open field was divided into three zones: an outer zone (20 cm wide), an inner zone (20 cm wide), and a center zone (40 x 40 cm; L x W). The following behaviors were analyzed: total distance traveled, time spent moving, latency to enter the inner and center zones, distance traveled in each zone, and duration in each zone. The open field was cleaned with a Nolvasan solution between animals.

### Elevated plus maze test

The elevated plus maze test was conducted as described previously [39]. The test apparatus consisted of four black polypropylene arms (Coulbourn Instruments, Whitehall, PA). The two “open” arms had 0.5 cm ledges and the two “closed” arms had 30 cm walls. The open arms were placed opposite of each other. The arms were 10 cm wide, 50 cm long, and were placed on 55 cm tall acrylic legs. Testing occurred in a quiet, dimly lit (75 lux) room. At the beginning of each test the animals were placed in the center of the apparatus facing an open arm. The animals were allowed to explore the apparatus for 5 min. The behavior of the animals was recorded with a monochrome CCD camera that was mounted above the elevated plus maze. The video signal was digitized with a frame grabber and then stored and analyzed with EthoVision XT 8.5 software (Noldus Information Technology, Leesburg, VA). The elevated plus maze was divided into 5 zones (two open arms, two closed arms, and center). The following behaviors were analyzed: distance traveled (open arms, closed arms, center area, and total), time moving, duration in each zone (open arms, closed arms, and center area), and number of open and closed arm entries. It was considered an open arm entry when the center of the rat was in one of the open arms. The apparatus was cleaned with a Nolvasan solution between animals.

### Somatic withdrawal signs

Rats were observed for 10 min in a Plexiglas observation chamber (25 × 25 × 46 cm; L x W x H). The rats were first habituated to the observation chamber for 5 min per day on three consecutive days prior to testing. The following somatic withdrawal signs were then recorded during testing: body shakes, cheek tremors, eye blinks, forepaw fluttering, gasps, genital licks, grooming, head shakes, ptosis, teeth chattering, writhes, and yawns [36, 40, 41]. Ptosis was counted once per minute if present continuously. The total number of somatic signs was defined as the sum of the individual occurrences of each behavior. For the final analyses some signs were grouped. Abdominal constrictions included gasps and writhes, shakes included head shakes and body shakes, and facial fasciculations include cheek tremors, chews, and teeth chattering. The somatic signs were recorded during the experiment by an experienced observer who was blind to the treatment conditions.

### Statistics

All the experiments were analyzed with the appropriate ANOVA and significant main effects and interaction effects are reported. Main effects of treatments, interaction effects between treatments, and interaction effects between treatments and time were investigated. In all statistical analyses, significant results in the ANOVAs were followed by Bonferroni’s post hoc comparisons to determine which groups differed from each other. The outcomes of the post hoc tests are reported in the tables and figures. P values less than 0.05 were considered significant.

In experiment 1, the effect of cannabis smoke on horizontal and vertical beam breaks in the small open field, behavioral parameters in the large open field, distance traveled in the elevated plus maze, and body weight gain were analyzed using two-way ANOVAs with smoke treatment as a between-subjects factor and time as a within-subjects factor. The effect of rimonabant on smoke-induced changes in beam breaks was analyzed using a three-way ANOVA with smoke treatment as a between-subjects factor and drug treatment (anandamide vs. vehicle) and time as within-subjects factors. The effect of cannabis smoke and rimonabant on the total number of somatic signs was analyzed using a two-way ANOVA with smoke treatment as a between-subjects factor and drug (rimonabant vs. vehicle) as a within-subjects factor. Behavioral parameters in the elevated plus maze test (except total distance traveled) and individual somatic withdrawal signs were analyzed with nonparametric statistical tests. The Wilcoxon signed-rank test was used for paired samples and the Mann-Whitney U test for independent samples.

In experiment 2, the effects of anandamide on beam breaks in the small open field test and behavioral parameters in the large open field test were analyzed using two-way repeated measures ANOVAs, with anandamide dose as a between-subjects factor and time as a within-subjects factor. The effect of rimonabant and anandamide on beam breaks in the small open field test was analyzed using three-way ANOVAs with anandamide and rimonabant treatments as between-subjects factors and time as a within-subjects factor.

In experiment 3, the effects of cannabis smoke exposure on anandamide-induced changes in beam breaks in the small open field were analyzed using a three-way ANOVA with smoke treatment (cannabis smoke vs. air) and drug treatment (anandamide vs. vehicle) as between-subjects factors and time as a within-subjects factor.

The data were analyzed with IBM SPSS Statistics version 22 and GraphPad Prism version 6.

## Results

### Experiment 1. Effect of cannabis smoke on exploratory behavior and development of dependence

#### Cannabis smoke and small open field

**Fig 2:**
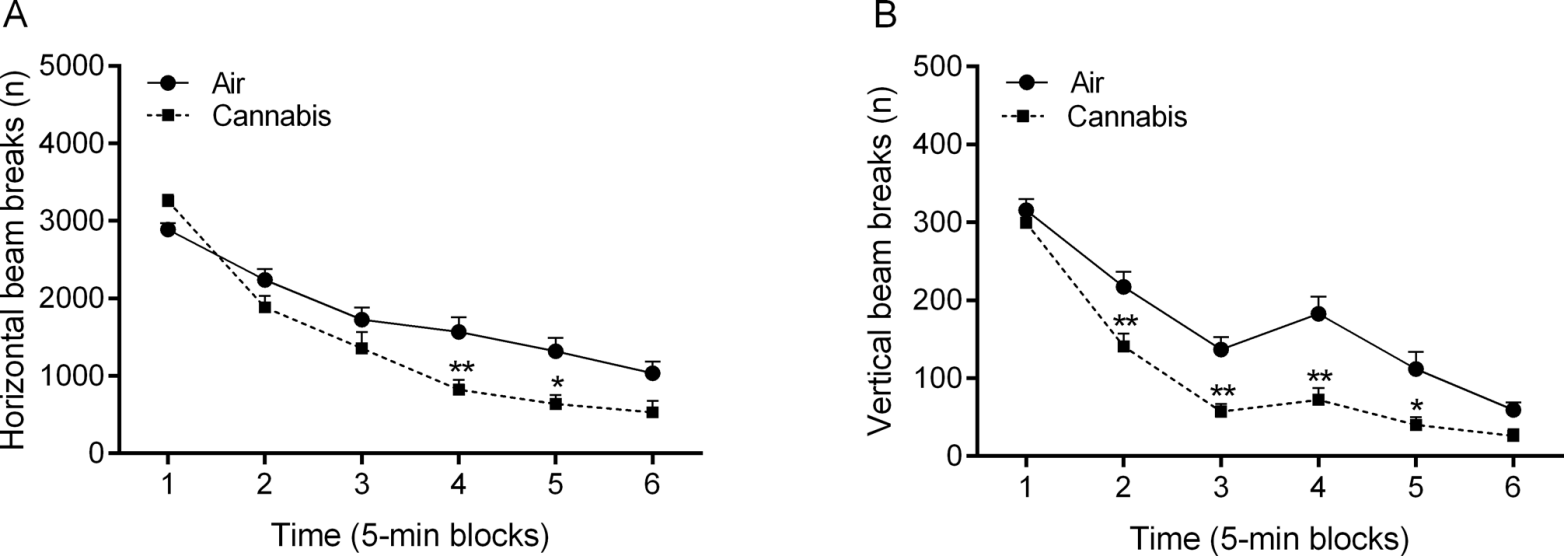
Cannabis smoke exposure decreases locomotor activity and rearing in the small open field. The rats were tested in the small open field immediately after cannabis smoke exposure and horizontal (A) and vertical beam breaks (B) were assessed. Asterisks (* p<0.05, ** p<0.01) indicate a significant difference from the air group. N = 10 per group. Abbreviation: Air, air-control group. Data expressed as means ± SEM.

The rats were exposed to cannabis smoke or air for 8 weeks and during this period both groups gained the same amount of weight (*time*: F7,126 = 882.62, p<0.0001, S1 Fig, S1 Table). Before the onset of these cannabis smoke exposure sessions, the air-control rats and the cannabis rats were tested in the small open field for 30 min. There were no differences in the total number of horizontal or vertical beam breaks between the air-control rats and the cannabis rats before the onset of the smoke exposure sessions (S2 Table). In both groups, there was an *effect of time* on horizontal (F5,90 = 93.16, p<0.0001) and vertical beam breaks (F5,90 = 34.33, p<0.0001, S2 Fig). This indicates that exploration decreased over time (6 blocks of 5 min each). After exposure to cannabis smoke, the rats were tested in the small open field for the second time (2 weeks after the first open field test). There was an effect of test (test 1 vs. test 2) on horizontal beam breaks (F1,9 = 6.43, p<0.03), but not on vertical beam breaks in the air-control rats (S2 Table). The number of horizontal beam breaks was slightly lower (10%) in the second compared to the first test (S2 Table). The effect of cannabis smoke on behavior in the small open field test is depicted in Fig 2 and S2 Table. There was an *effect of time* on horizontal (F5,90 = 85.15, p<0.0001, Fig 2A) and vertical beam breaks (F5,90 = 119.78, p<0.0001, Fig 2B). Furthermore, there was an *effect of cannabis smoke exposure* on horizontal (F1,19 = 7.12, p<0.05) and vertical beam breaks (F1,19 = 14.11, p<0.001), which was due to a smoke-induced decrease in beam breaks (S2 Table). In addition, there was a *time x smoke exposure interaction* for horizontal (F5,90 = 4.72, p<0.001) and vertical beam breaks (F5,90 = 3.90, p<0.01). The post hoc analyses showed that exposure to cannabis smoke decreased horizontal (Fig 2A) and vertical beam breaks (Fig 2B). We also detected a *time x smoke exposure interaction* for the total distance traveled (F5,90 = 6.90, p<0.0001), indicating that cannabis smoke exposure increased locomotor activity at the beginning of the test and decreased locomotor activity at the end of the test (S3 Fig).

#### Cannabis smoke, Δ9-THC levels, and development of dependence

**Fig 3:**
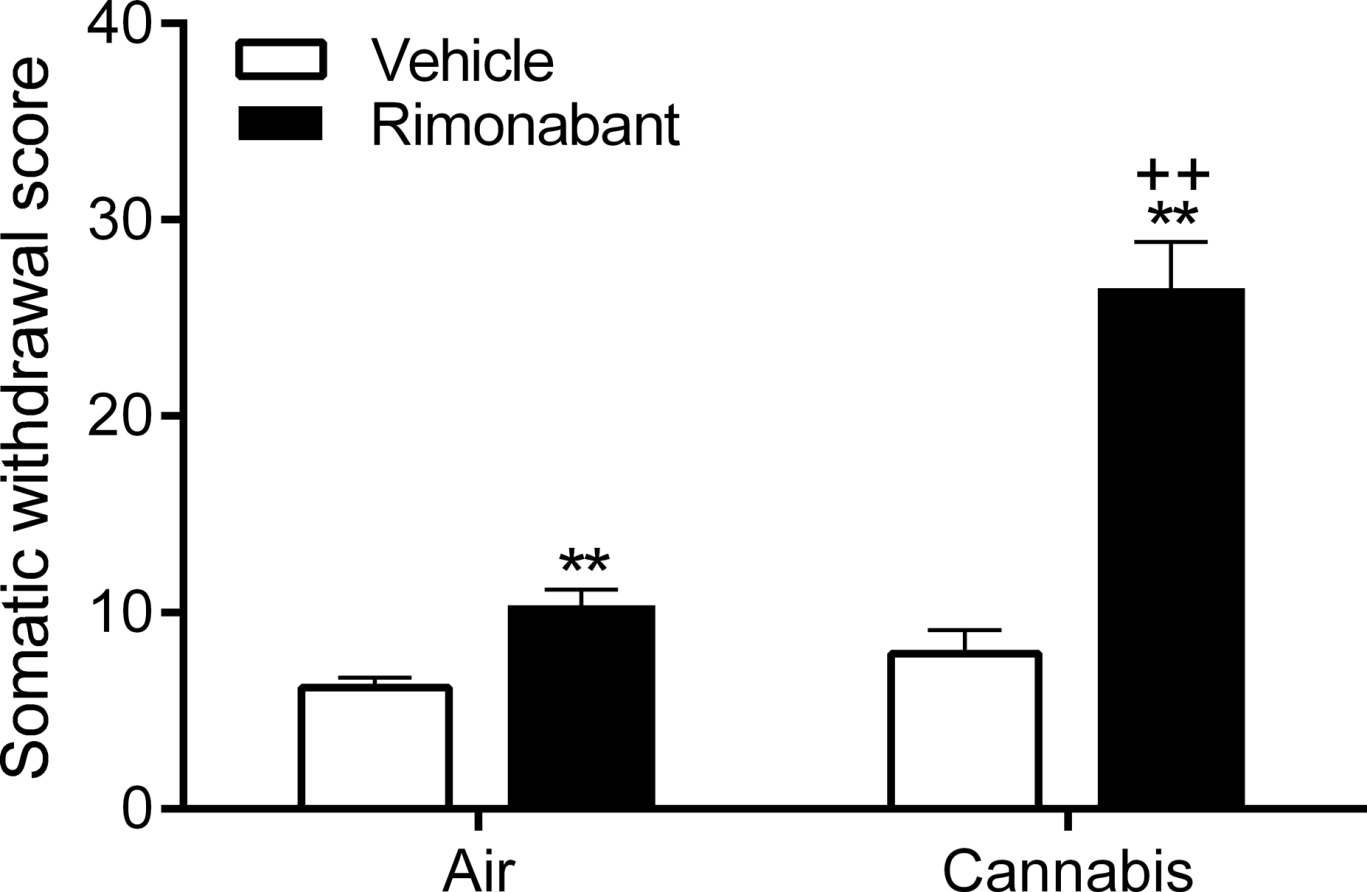
Increase in somatic withdrawal signs in rats exposed to cannabis smoke. The CB_1_ receptor antagonist rimonabant induces more somatic withdrawal signs in the cannabis rats than the air exposed rats. Asterisks (** p<0.01) indicate increased somatic withdrawal signs compared to the corresponding vehicle group. Plus signs (++ p<0.01) indicate increased somatic withdrawal signs compared to the air-rimonabant group. N = 10 per group. Data expressed as means ± SEM.

The cannabis rats and air-control rats received vehicle or rimonabant and somatic withdrawal signs were recorded. There was an *effect of drug treatment* (rimonabant, F1,36 = 23.00, p<0.0001), *smoke treatment* (F1,36 = 35.14, P<0.0001), and a *drug x smoke treatment interaction* (F1,36 = 23.00, p<0.0001) for the total number of somatic withdrawal signs (Fig 3). This indicates that rimonabant induced somatic withdrawal signs in both the air-control rats and the cannabis rats, but to a greater extent in the cannabis rats than in the air-control rats. Nonparametric analyses of the individual somatic withdrawal signs indicated that rimonabant induced more eye blinks (p<0.01) and grooming (p<0.01) in cannabis rats compared to air-control rats (Mann-Whitney U Test, S3 Table). The cannabis rats also displayed more eye blinks (p<0.05), forepaw fluttering (p<0.05), grooming (p<0.01), ptosis (p<0.05), and shakes (p<0.05) after treatment with rimonabant compared to treatment with vehicle (Wilcoxon signed-rank test). Rimonabant also induced more grooming (p<0.05) and shakes (p<0.05) compared to vehicle in the air-control group (Wilcoxon signed-rank test), but it should be noted that the cannabis rats displayed significantly more grooming after treatment with rimonabant than the air-control rats.

Blood samples were collected in week 2 and 4 immediately after cannabis smoke exposure. Plasma Δ9-THC levels were 224.9 ± 3.1 ng/ml (n = 10) and 222.9 ± 6.4 ng/ml (n = 10) in week 2 and 4, respectively.

#### Role of CB1 receptors in cannabis smoke-induced changes in small open field behavior

The rats were tested in the small open field approximately 4 h after smoke exposure. Before the administration of rimonabant, the animals were placed in the small open field and baseline activity was recorded for 30 min (S4 Table). During this 30-min period there was an *effect of smoke exposure* on horizontal (F1,18 = 4.79, p<0.05) and vertical beam breaks (F1,18 = 5.52, p<0.05), which was due to lower number of beam breaks in the cannabis rats. During the baseline period, the rats made slightly fewer horizontal beam breaks during the second compared to the first test session (F1,18 = 10.81, p<0.01).

**Fig 4:**
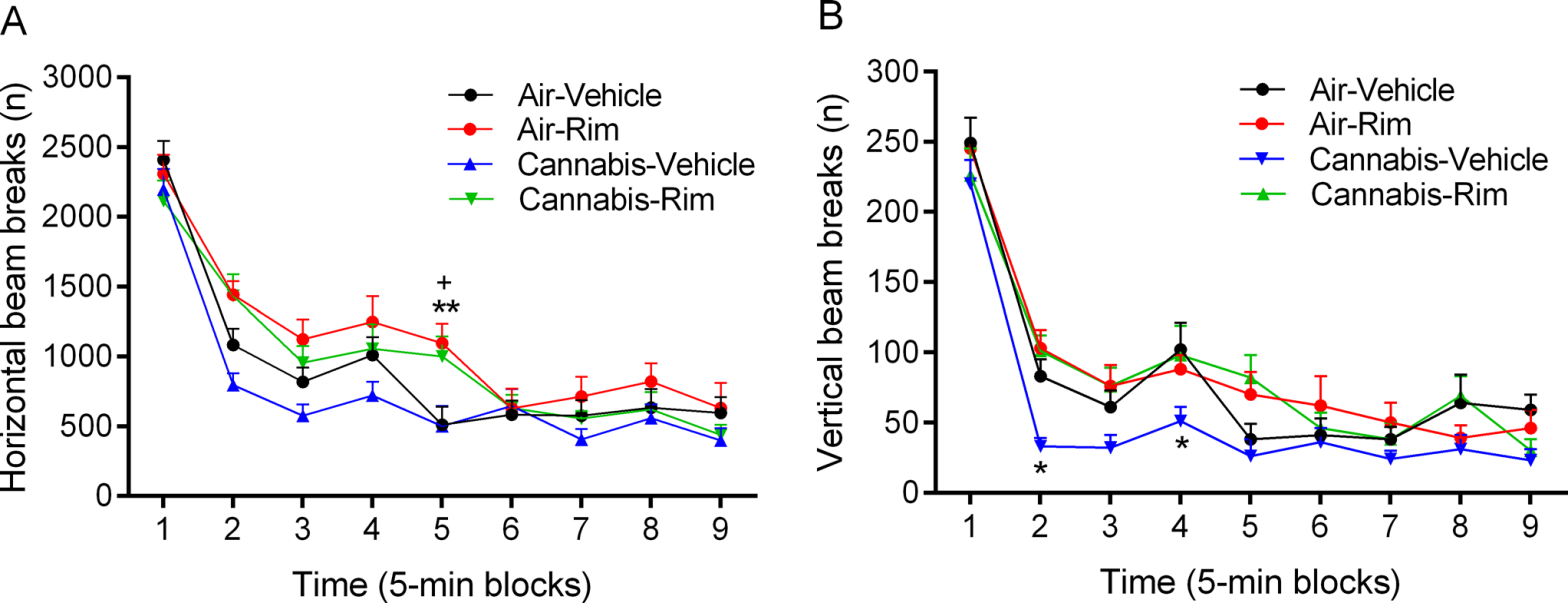
Blockade of CB_1_ receptors increases locomotor activity and prevents the cannabis smoke-induced decrease in rearing. The effect of rimonabant and cannabis smoke on horizontal (A) and vertical beam breaks (B) was investigated 4 h after smoke exposure. A: Plus signs (+ p<0.05, cannabis-rimonabant group) and asterisks (** p<0.01, air-rimonabant group) indicate an increase in horizontal beam breaks compared to the air-vehicle group. B: Asterisks (* p<0.05) indicate a decrease in vertical beam breaks compared to the air-vehicle group. N = 10 per group. Abbreviations: Air, air-control group; Rim, rimonabant. Data expressed as means ± SEM.

After the administration of rimonabant the rats were tested for an additional 45 min in the small open field (S4 Table). In order to correct for test effects, one half of the rats received vehicle during the first test and the other half received rimonabant. There was an *effect of time* (9 blocks of 5 min each) on horizontal (F8,144 = 81.58, p<0.0001, Fig 4A) and vertical beam breaks (F8,144 = 107.91, p<0.0001, Fig 4B), which indicates a decrease in exploration over time. In addition, there was an *effect of drug treatment* (vehicle vs. rimonabant) on horizontal (F1,18 = 18.45, p<0.0001) and vertical beam breaks (F1,18 = 7.26, p<0.05). We also detected a *drug treatment x time interaction* for horizontal beam breaks (F8,144 = 3.49, p<0.001). The post hoc analyses showed that rimonabant slightly increased horizontal beam breaks in the air and cannabis rats (Fig 4A) and prevented the cannabis smoke induced decrease in vertical beam breaks (Fig 4B).

#### Cannabis smoke and elevated plus maze test and large open field

**Fig 5:**
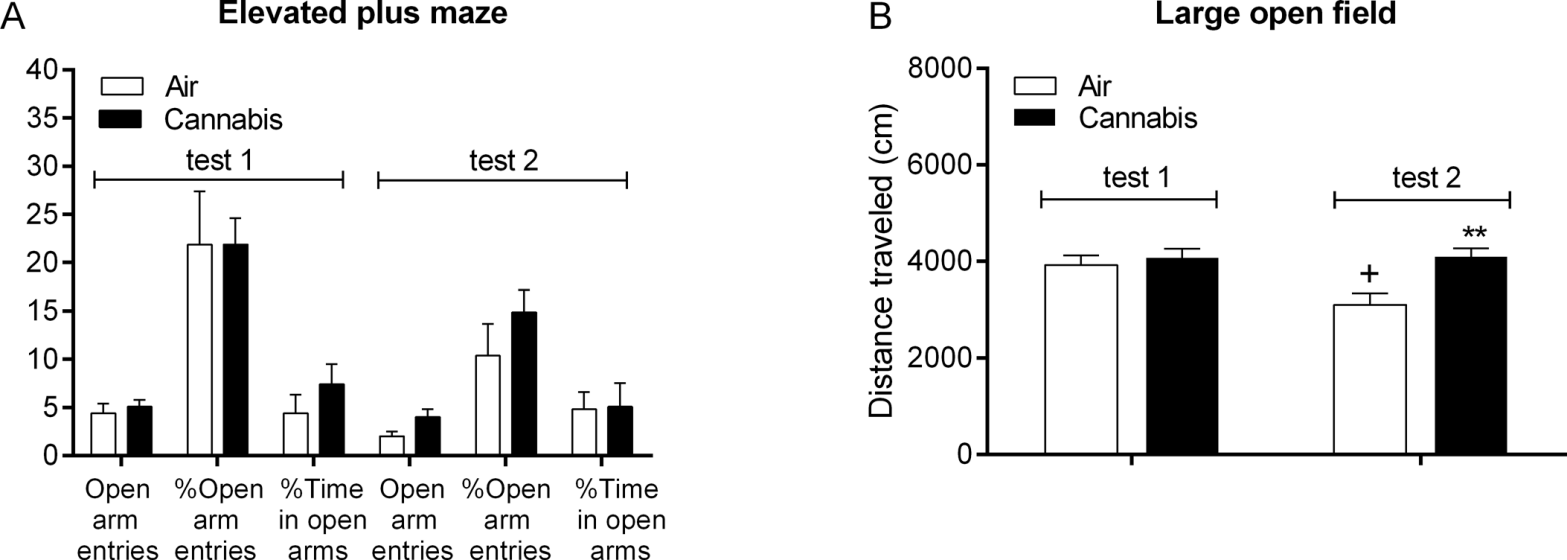
Behavior in the elevated plus maze and large open field after cannabis smoke exposure. The effect of cannabis smoke on the number of open arm entries, % open arm entries, % time in the open arms in the elevated plus maze (A) and distance traveled in the large open field (B) was investigated 48 h (test 1) and immediately after smoke exposure (test 2). B: Asterisks (** p<0.01) indicate an increase in the distance traveled compared to the air group during the second test, and the plus sign (+ p<0.05) indicates a decrease in the distance traveled compared to the same group during the first test. N = 10 per group. Abbreviation: Air, air-control group. Data expressed as means ± SEM.

The rats were tested twice in the elevated plus maze test: 48 h after cannabis smoke exposure, and the following day immediately after cannabis smoke or air exposure (Fig 5A, S5 Table). There was an *effect of time* (test 1 vs. test 2) on total distance traveled (F1,18 = 28.61, p<0.0001) and there was a *time x smoke exposure interaction effect* for the total distance traveled (F1,18 = 5.95, p<0.05, S5 Table). A separate analysis for each zone indicated that there was an *effect of time* (F1,18 = 16.06, p<0.001) and a *time x smoke exposure interaction effect* (F1,18 = 6.17, p<0.05) for the distance traveled in the closed arms (S5 Table). In addition, there was an *effect of time* (F1,18 = 36.35, p<0.0001) on the distance traveled in the center area. Bonferroni post hoc analyses indicated that when the rats were tested for the first time in the elevated plus maze test (48 h post cannabis smoke) there was no difference in total distance traveled between the air-control and cannabis rats, but during the second test the cannabis rats traveled a greater distance than the air-control rats. This increase in activity in the cannabis rats was due to an increase in exploration of the closed arms. The post hoc analysis indicated that during the second test the cannabis rats traveled a greater distance in the closed arms than the air-control rats, but there was no difference in the distance traveled in the open arms. During the second test, both the air-control rats and the cannabis rats traveled a greater distance in the center area compared to the first test. However, because of the relatively small distance traveled in the center area, this only marginally contributed to the total distance traveled. Additional nonparametric analyses showed that during the second test the cannabis rats spent more time moving than the air-control rats (Mann-Whitney U Test, p<0.05, S5 Table). Furthermore, during the second test the cannabis rats spent more time in the center area than during the first test (Wilcoxon signed-rank test, p<0.05) and made more entries into the closed arms than during the first test (Wilcoxon signed-rank test, p<0.05). During the second test the air-control rats made fewer open arm entries than during the first test but this remained stable for the cannabis rats (Wilcoxon signed-rank test, p<0.05). These data indicate that acute exposure to cannabis smoke increased exploration of the closed arms and attenuated the decrease in exploration of the open arms.

The rats were tested twice in the large open field (5 min): 48 h after smoke or air exposure and the following day immediately after smoke or air exposure (Fig 5B, S6 Table). There was an *effect of time* (test 1 vs. test 2) on total time moving (F1,18 = 20.83, p<0.0001) and distance traveled in the inner zone (F5,90 = 89.36, p<0.05). There was an *effect of cannabis smoke exposure* on total distance traveled (Fig 4B; F1,18 = 7.13, p<0.05), total time moving (F1,18 = 7.11, p<0.05), distance traveled in outer zone (F1,18 = 4.44, p<0.05), time in inner zone (F1,18 = 5.06, p<0.05). There was also *a time x smoke exposure interaction* for the total time moving (F1,18 = 6.43, p<0.05). The post hoc analyses suggested that most of these effects were due to the effects of acute cannabis smoke on exploration during the second test (Fig 5B). During the first test there were no differences between the air-control and cannabis rats. During the second test the air-control rats traveled less than during the first test but this did not change for the cannabis rats.

### Experiment 2. Effect of anandamide on exploratory behavior

#### Anandamide and small open field

**Fig 6:**
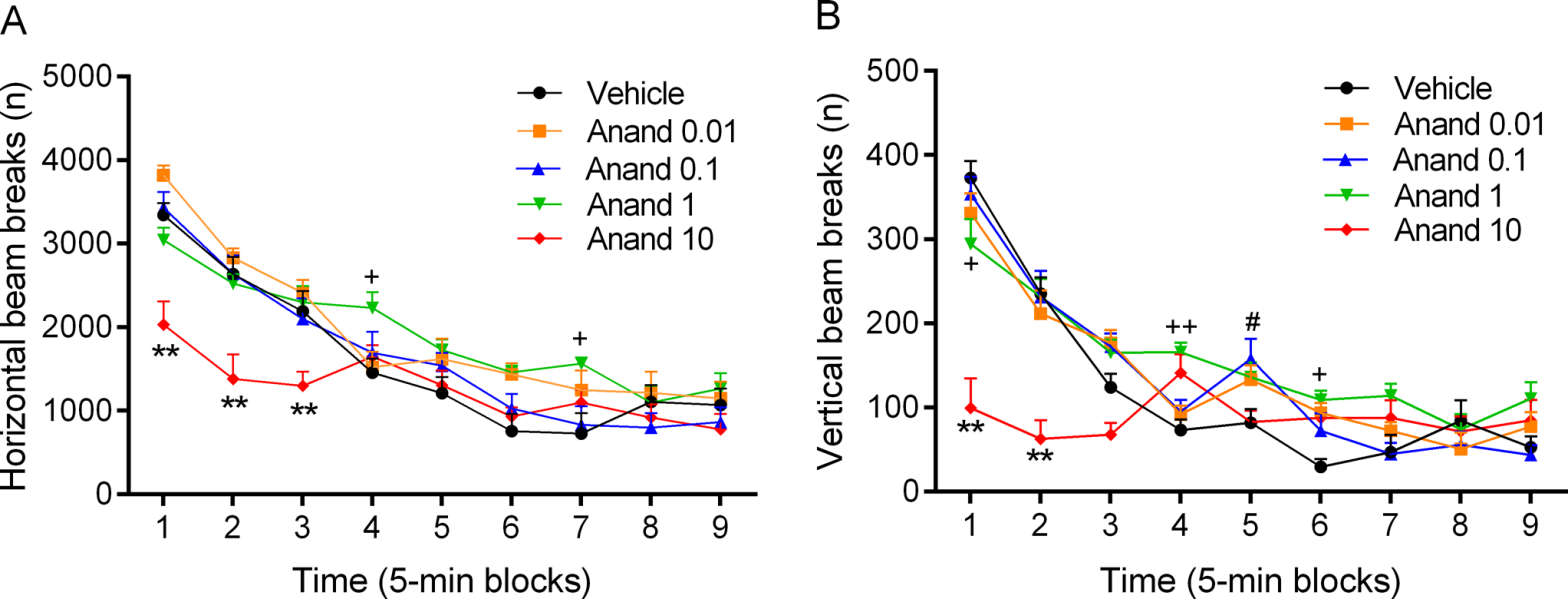
Anandamide decreases locomotor activity and rearing in small open field. Immediately after the rats received anandamide they were placed in the small open field and horizontal (A) and vertical beam breaks (B) were assessed. Pound sign (0.1 mg, # p<0.05), plus signs (1 mg, + p<0.05, ++ p<0.01), and asterisks (10 mg, * p<0.05, ** p<0.01) indicate an increase or decrease in horizontal or vertical beam breaks compared to the vehicle group. N = 10 per group. Abbreviation: Anand, anandamide. Data expressed as means ± SEM.

The rats received acute injections of anandamide and were then placed in the small open field (45 min, S7 Table). There was an *effect of time* on horizontal (F8,360 = 89.29, p<0.0001, Fig 6A) and vertical beam breaks (F8,360 = 74.95, p<0.0001, Fig 6B). There was an *effect of drug treatment* (anandamide) on horizontal (F4,45 = 5.55, p<0.001) and vertical beam breaks (F4,45 = 8.36, p<0.0001). There was also a *time x drug treatment interaction* for horizontal (F32,360 = 3.14, p<0.0001) and vertical beam breaks (F32,360 = 6.69, p<0.0001). The post hoc analysis revealed that low doses of anandamide increased horizontal (1 mg/kg, Fig 6A) and vertical beam breaks (0.1 and 1 mg/kg, Fig 6B). The 10 mg/kg dose decreased both horizontal and vertical beam breaks. The main finding of this experiment was that low doses of anandamide increase and a high dose decreases exploration of the small open field.

#### Anandamide and large open field

**Fig 7:**
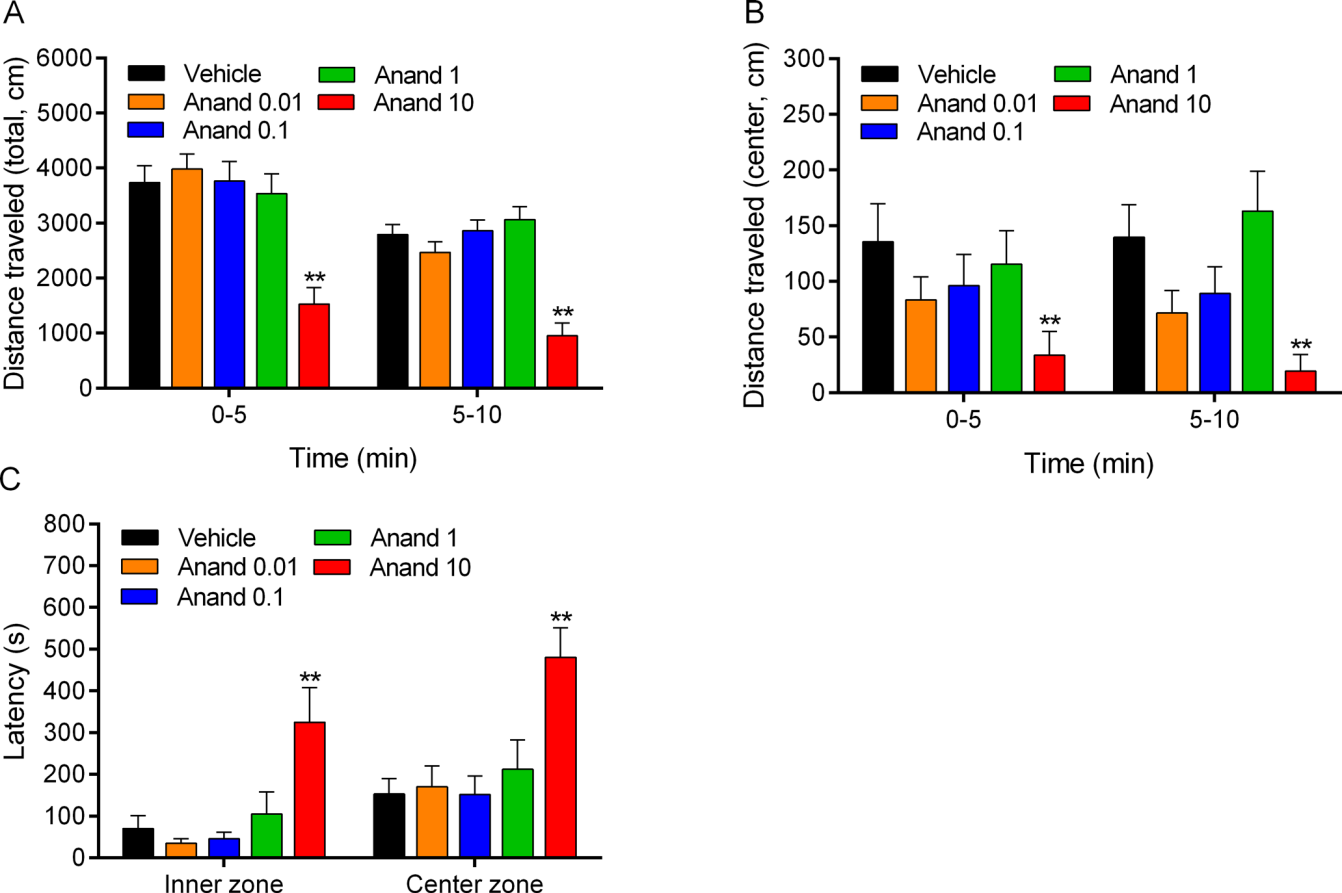
Anandamide decreases exploratory behavior in the large open field. Immediately after the rats received anandamide they were placed in the large open field and the total distance traveled (A), center distance (B), and center latency (C) were assessed. Asterisks (** p<0.01) indicate a decrease in the distance traveled or increase in the latency compared to the vehicle group. N = 10 per group. Abbreviation: Anand, anandamide. Data expressed as means ± SEM.

The rats received acute injections of anandamide and were then placed in the large open field (10 min, S8 Table). There was an *effect of time* (0–5 vs. 5–10 min) on the total distance traveled (F1,45 = 53.22, p<0.0001, Fig 7A), total time moving (F1,45 = 45.66, p<0.0001), and distance traveled in the outer zone (F1,45 = 69.33, p<0.0001), indicating that exploration of the large open field decreased over time (S8 Table). A high dose of anandamide (10 mg/kg) induced a strong decrease in exploration of the large open field and this was reflected in an *effect of drug treatment* on the total distance traveled (F4,45 = 13.37, p<0.0001), total time moving (F4,45 = 17.46, p<0.0001), distance traveled in the outer zone (F4,45 = 16.51, p<0.0001), distance traveled in the inner zone (F4,45 = 4.80, P<0.01), distance traveled in the center zone (F4,45 = 3.61, p<0.05, Fig 7B), time in the outer zone (F4,45 = 5.30, p<0.01), time in the inner zone (F4,45 = 5.39, p<0.01), time in the center zone (F4,45 = 3.61, p<0.05), latency to enter the center zone (F4,45 = 5.55, p<0.01, Fig 7C), and latency to enter the inner zone (F4,45 = 5.82, p<0.001). The main finding of this experiment is that a high dose of anandamide decreases locomotor activity in the large open field.

#### Role of CB1 receptors in anandamide-induced changes in small open field behavior

**Fig 8:**
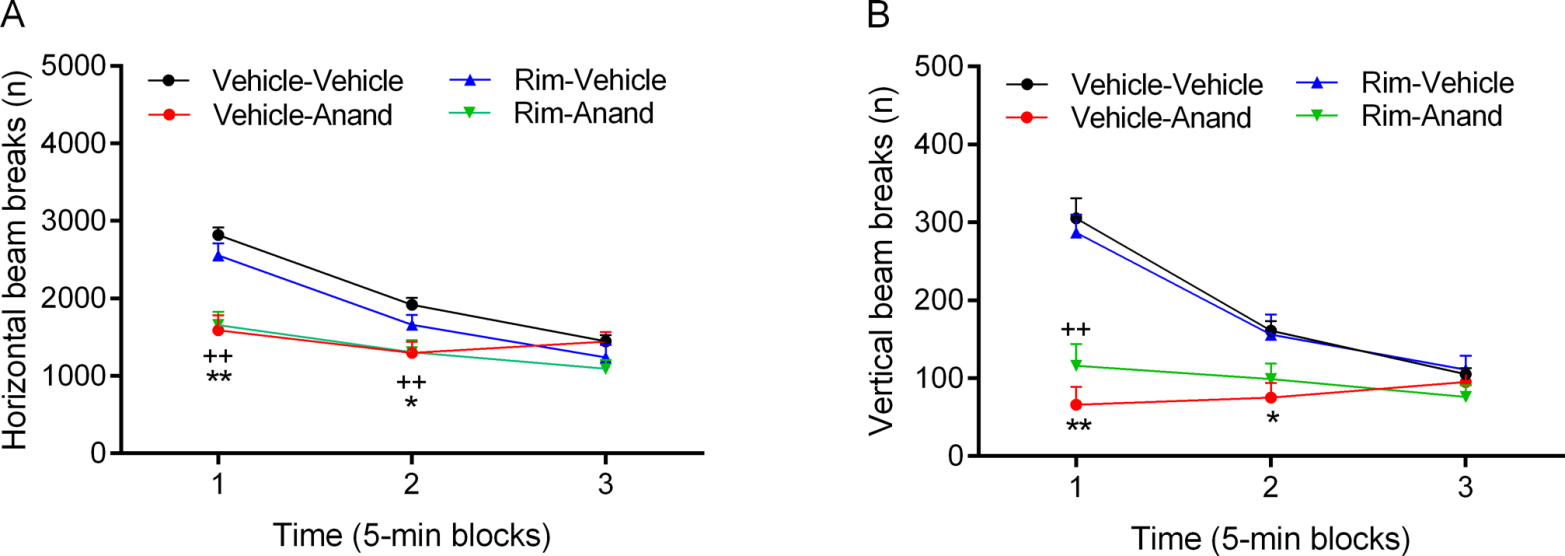
Blockade of CB_1_ receptors does not prevent the anandamide-induced decrease in locomotor activity and rearing. Rats were treated with rimonabant (5 mg/kg, ip) and anandamide (10 mg/kg, ip) and horizontal (A) and vertical beam breaks (B) were assessed in the small open field. Plus signs (++ p<0.01, rimonabant-anandamide group) and asterisks (* p<0.05, ** p<0.01, vehicle-anandamide group) indicate a decrease in horizontal and vertical beam breaks compared to the vehicle-vehicle group. N = 10–16 per group. Abbreviations: Anand, anandamide; Rim, rimonabant. Data expressed as means ± SEM.

The rats received rimonabant followed by anandamide or control treatments (4 groups) and were then placed in the small open field for 15 min (S9 Table). There was an *effect of time* on horizontal (F2,90 = 49.11, p<0.0001, Fig 8A) and vertical beam breaks (F2,90 = 41.11, p<0.0001, Fig 8B), such that exploration of the small open field gradually decreased over time. The ANOVA analyses revealed an effect of *drug treatment* (i.e., anandamide) on horizontal (F1,45 = 24.75, p<0.0001) and vertical beam breaks (F1,45 = 30.21, p<0.0001). These effects were due to the fact that anandamide decreased locomotor activity and rearing in the small open field. In addition, there was a *drug treatment* (*anandamide) x time interaction* for horizontal (F2,90 = 15.91, p<0.0001) and vertical beam breaks (F2,90 = 36.17, p<0.0001). This indicates that the effects of anandamide were time dependent and a close look at the data indicates that the effects of anandamide were most pronounced during the first 5 min of the small open field test. There were no main effects of the pre-treatment drug (rimonabant) or any pre-treatment drug (rimonabant) x drug (anandamide) interactions. This suggests that rimonabant alone did not affect open field behavior, nor did it influence the behavioral effects of anandamide. The post hoc comparisons indicated that anandamide decreased horizontal (Fig 7A) and vertical beam breaks for 10 min (Fig 7B), and that there were no significant differences between the anandamide rats treated with vehicle or rimonabant. The main finding of this experiment was that CB_1_ receptor blockade did not affect the anandamide-induced decrease in locomotor activity and rearing.

### Experiment 3. Effect of cannabis smoke on anandamide-induced changes in exploratory behavior in the small open field

**Fig 9:**
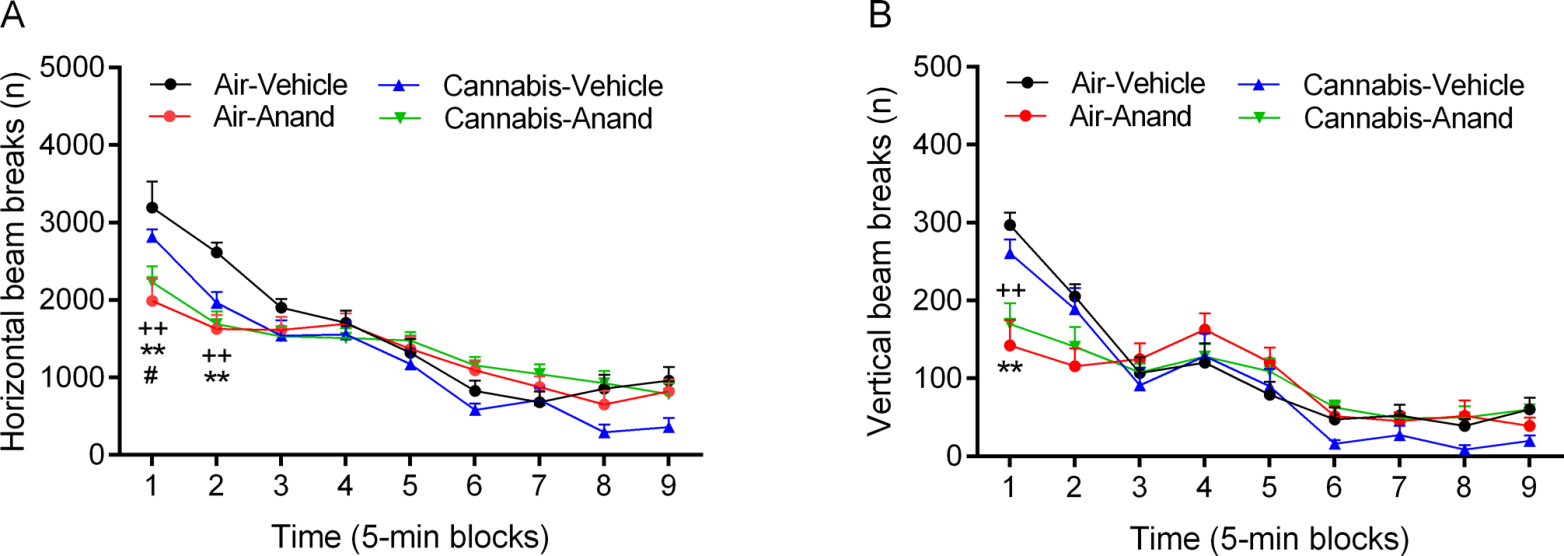
Chronic exposure to cannabis smoke does not prevent the anandamide-induced decrease in locomotor activity and rearing. Rats were exposed to cannabis smoke for 2 weeks and 24 h after the last smoke exposure session it was investigated if pre-exposure to smoke affected anandamide (10 mg/kg, ip) induced changes in horizontal (A) and vertical beam breaks (B) in the small open field. A, B: Plus signs (++ p<0.01, cannabis-anandamide group) and asterisks (** p<0.01, air-anandamide group) indicate a decrease in horizontal and vertical beam breaks compared to the air-vehicle group. A: Pound sign (# p<0.05, cannabis-vehicle group) indicates a decrease in horizontal beam breaks compared to the air-vehicle group. N = 10–16 per group. Abbreviations: Air, air-control group; Anand, anandamide. Data expressed as means ± SEM.

It was investigated if chronic exposure to cannabis smoke affects the response to anandamide in the small open field. Rats were exposed to cannabis smoke and 24 h after the last exposure they received anandamide and were tested in the small open field (45 min). There was an *effect of time* on body weight gain (F1,38 = 2223.12, p<0.0001) and there was an *effect of smoke treatment* on body weight gain (F1,38 = 11.42, p<0.002), such that the cannabis smoke exposed rats gained slightly less weight over the two weeks of exposure compared to the air-control rats (S1B Fig). In the small open field test (immediately after anandamide administration, S10 Table), there was an *effect of time* on horizontal (F8,288 = 85.98, p<0.0001, Fig 9A) and vertical beam breaks (F8,280 = 61.14, p<0.0001, Fig 9B). Exploration of the small open field decreased over time. There was a *time x drug treatment (anandamide) interaction* for horizontal (F8,288 = 10.06, p<0.0001) and vertical beam breaks (F8,288 = 10.75, p<0.0001), and a *drug treatment x smoke treatment interaction* for horizontal beam breaks (F1,36 = 4.81, p<0.05). Post hoc comparisons indicated that anandamide decreased horizontal beam breaks (Fig 9A) during the first 10 min and vertical beam breaks (Fig 9B) during the first 5 min in both the air-control and cannabis rats. This indicates that exposure to cannabis smoke does not affect the anandamide-induced decrease in locomotor activity and rearing.

## Discussion

The present studies investigated the effects of cannabis smoke exposure on exploratory behavior and the development of cannabis dependence in rats. The studies showed that cannabis smoke increased locomotor activity when the rats were tested immediately after smoke exposure, but not when tested 4 h, 24 h or 48 h after cannabis smoke exposure. Exposure to cannabis smoke also led to a decrease in rearing which was observed immediately and 4 h after smoke exposure, but not 24 h after smoke exposure. The smoke-induced decrease in rearing was blocked by treatment with the CB_1_ receptor antagonist rimonabant. Chronic exposure to cannabis smoke also led to dependence as indicated by rimonabant-induced somatic withdrawal signs. A high dose of the endogenous cannabinoid anandamide decreased locomotor activity and rearing, but this was not affected by pretreatment with rimonabant. In contrast to a high dose of anandamide, low doses of anandamide increased locomotor activity in the small open field. Furthermore, chronic exposure to cannabis smoke did not affect the anandamide-induced decrease in locomotor activity and rearing. These studies indicate that cannabis smoke exposure has bidirectional effects on locomotor activity (brief increase followed by decrease), induces dependence, but does not induce tolerance to the locomotor suppressant effects of anandamide.

Blood samples were collected at two time points (week 2 and week 4) immediately after smoke exposure to assess serum Δ9-THC levels. Exposure to cannabis smoke led to serum Δ9-THC levels of approximately 225 ng/ml. One study with humans reported plasma Δ9-THC levels of 250 ng/ml immediately after smoking a cannabis cigarette that contained 3.55% Δ9-THC [42]. In another study with mice, cannabis smoke was generated by burning 100 mg of 3.46% Δ9-THC cannabis over a 5-min period [43]. Blood and brains were collected 20 min after smoke exposure and Δ9-THC levels were 241 ng/ml and 256 ng/ml in plasma and brain, respectively. This suggests that after the inhalation of cannabis smoke there is a strong correlation between plasma and brain Δ9-THC levels and therefore that plasma Δ9-THC levels might be indicative of brain levels. Delta9-THC levels in our study were in line with those obtained in previous work, indicating that we have successfully established an animal model of cannabis smoke exposure in humans.

In order to determine if exposure to cannabis smoke leads to the development of dependence the rats were treated with the CB_1_ receptor antagonist rimonabant or vehicle, and somatic withdrawal signs were recorded. Rimonabant induced more somatic signs in the cannabis rats than in the air-control rats. This observation is in line with previous studies that investigated precipitated somatic withdrawal signs in Δ9-THC-treated rodents [27, 44, 45]. Although rimonabant induced a small increase in ptosis, shakes, and forepaw fluttering in the cannabis rats, the large increase in the total number of somatic signs in this group was mainly due to an increase in eye blinks and grooming. It is interesting to note that precipitated cannabis withdrawal induces a large increase in grooming behavior. Previous studies have shown that rats display extensive grooming behavior in response to mild stressors or after the administration of stress peptides including adrenocorticotropic hormone (ACTH) [46]. Grooming is often considered a displacement activity that animals display in response to stress, conflict, or frustration [47, 48]. Therefore, the increase in grooming behavior in rats undergoing cannabis withdrawal might be indicative of emotional distress. Rimonabant also induced a small increase in grooming and shakes in the air-control rats. This observation is in line with other studies that reported that rimonabant increases grooming and shakes in drug naïve rats [49, 50]. It might be possible that blockade of CB_1_ receptors induces a mildly aversive state that leads to an increase in these behaviors [51, 52]. Taken together, these studies suggest that the chronic cannabis smoke exposure model used here leads to adaptations in CB_1_ receptor function that are indicative of the development of cannabis dependence.

The present study investigated the effects of cannabis smoke on the behavior of rats in the small and large open field test and the elevated plus maze test. In addition, the effect of anandamide on the behavior of rats in the small and large open field test was investigated. The small open field has been widely used to assess the stimulant-like effects of drugs, whereas the large open field and elevated plus maze can be used to assess locomotor activity as well as anxiety-like behavior. Stressful stimuli and anxiogenic drugs decrease the amount of time spent in the center of the large open field or the open arms of the elevated plus maze, and anxiolytic drugs have the opposite effect [53, 54]. In the present studies we found that the cannabis rats displayed an increase in locomotor activity during the first 5-min of the small open field test and during the 5-min large open field test and elevated plus maze test. This increase in locomotor activity was only detected when the rats were tested immediately after the smoke exposure. When the rats were tested 4 h, 24 h, or 48 h after smoke exposure there was no difference in locomotor activity between the cannabis rats and the air-control rats. The cannabis smoke-induced increase in locomotor activity might model the acute energizing and uplifting effects of cannabis smoke that have been reported in human cannabis users [55, 56].

Some conflicting findings have been reported with regard to the effects of Δ9-THC on locomotor activity in rodents. A number of initial studies reported that Δ9-THC dose-dependently decreases locomotor activity [57–59]. However, a recent study suggests that Δ9-THC has biphasic effects, with low doses increasing and high doses decreasing locomotor activity [60]. The discrepancy between these studies might be due to differences in sampling duration or the time points at which locomotor activity was assessed. Katsidoni and colleagues [60] found that a low dose of Δ9-THC increased locomotor activity 1 to 2 h after drug administration, whereas the other studies recorded locomotor activity for a brief period immediately after drug administration [57–59]. Several studies have investigated the effects of cannabis smoke on locomotor activity in mice, and have found that smoke from both cannabis and “placebo cannabis” (containing negligible amounts of Δ9-THC) decreases locomotor activity to a similar degree [61, 62]. In these studies, the mice were restrained while being exposed to smoke and locomotor activity in the cannabis smoke-exposed mice did not differ from locomotor activity in mice in the restraint/air-control group [61]. This suggests that in these studies the decrease in locomotor activity was at least partly due to stress caused by the combination of restraint and smoke exposure. In contrast, a study that investigated the effects of cannabis smoke on freely moving mice reported that exposure to smoke induces a brief increase in locomotor activity (1–3 min) followed by a decrease in locomotor activity [63]. Inhalation of cannabis smoke with Δ9-THC has also been shown to induce a brief period of hyperactivity followed by hypoactivity in rats when compared to rats exposed to smoke from placebo cigarettes [64]. These studies suggest that the effects of cannabis smoke on locomotor activity may depend on the exposure conditions (i.e., restraint vs. no restraint), Δ9-THC level, the time between smoke exposure sessions, and the behavioral test. Our results resemble those of studies that investigated the effects of the inhalation of Δ9-THC containing cannabis smoke in freely moving animals. It should be noted that in the present study we investigated the effect of one dose of cannabis smoke in freely moving rats and others have investigated the effects of several doses in restraint rats or mice (nose-only exposure)[61, 62, 64]. It is very likely that lower or higher doses than those used in the present study would have had different effects on open field behavior. Therefore, additional studies in which freely moving animals are exposed to multiple levels of cannabis smoke are needed to better understand the effects of cannabis smoke on exploratory behavior.

Initial studies reported that anandamide decreases locomotor activity [26, 65], but a more recent study that used a wide range of anandamide doses showed that low doses increase locomotor activity and high doses decrease locomotor activity [66]. In the present study, we showed that a low dose of anandamide (0.01 mg/kg) slightly, but not significantly, increased locomotor activity during the first 5 min of the small open field test and a somewhat higher dose (1 mg/kg) increased locomotor activity at later time points. The highest dose (10 mg/kg) induced a large decrease in locomotor activity during the first 10 min of the test. We also showed that a high dose of anandamide induced a dramatic decrease in rearing and that low doses increased rearing from 15–30 min after anandamide administration. The 1 mg/kg anandamide dose also increased the total amount of rearing (S7 Table, vertical beam breaks). These findings are in line with a study in mice that showed that low doses of anandamide increase rearing and high doses decrease rearing [66]. Interestingly, we did not detect an anandamide-induced increase in locomotor activity in the large open field. This might be due to the fact that large open spaces are more fear-provoking than small open spaces. The small open field test is widely used to assess the psychomotor effects of drugs whereas the large open field is mostly used to assess the anxiolytic or anxiogenic effects of drugs [67–69]. Therefore, our results suggest that low doses of anandamide may induce a small increase in locomotor activity but have no anxiolytic-like effects. This is also supported by the fact that low doses of anandamide did not decrease the latency to enter the center zone of the large open field and did not affect the amount of time spent in the center of the large open field.

Exposure to cannabis smoke induced a dramatic decrease in rearing that was observed when the rats were tested either immediately or 4 h after smoke exposure. This decrease in rearing was most apparent during the first 20 min of the small open field test. After about 20 min this effect dissipated due to a strong decrease in rearing in the air-control animals. The decrease in rearing was not detected when the rats were tested 24 h after cannabis smoke exposure. This suggests that the cannabis-induced decrease in rearing is temporary and returns to baseline levels after most of the Δ9-THC has been metabolized. Delta-9-THC levels decline in a biphasic fashion in rats, with a rapid decline immediately after administration (t_1/2_ = 30 min) followed by a very slow decline in remaining Δ9-THC levels (t_1/2_ = 16 h)[70]. The cannabis smoke-induced decrease in rearing was detected at time points at which locomotor activity was not affected. Therefore, this suggests that the decrease in rearing was not due to a general decrease in exploration or to sedative effects of the cannabis smoke. The cannabis smoke-induced decrease in rearing was completely blocked by administering the CB_1_ receptor antagonist rimonabant before the small open field test. This clearly demonstrates that the effects of cannabis smoke on rearing are mediated via the CB_1_ receptor. Very high levels of CB_1_ receptors have been detected in brain areas that play a role in motor function, including the basal ganglia and cerebellum [20, 21]. Systemic administration of Δ9-THC [71] as well as direct administration of Δ9-THC or CB_1_ receptor agonists into the cerebellum have been shown to impair motor coordination on the rotarod test [72, 73]. Therefore, it is possible that the cannabis smoke-induced decrease in rearing is due to a CB_1_ receptor-dependent impairment in balance and motor coordination.

It is interesting to note that rimonabant prevented the cannabis smoke-induced decrease in rearing but did not affect the anandamide-induced decrease in rearing or locomotor activity. This is in line with studies showing that rimonabant attenuates Δ9-THC but not anandamide-induced immobility in mice [74, 75]. It should be noted that although rimonabant does not affect the anandamide-induced decrease in locomotor activity, it has been shown to block the effects of anandamide on vas deferens contractions [17], blood pressure responses [76], and long term potentiation in the hippocampus [77]. At this point it is not completely clear why rimonabant inhibits the effects of cannabis smoke and Δ9-THC but not those of anandamide on open field behavior. However, it has been suggested that Δ9-THC and anandamide interact differently with the CB_1_ receptor and this might partly explain why rimonabant diminishes the effects of Δ9-THC but not those of anandamide on behavior [75]. It has also been reported that anandamide, but not Δ9-THC, is a transient receptor potential vanilloid type 1 (TRPV1) receptor agonist [75, 78]. The TRPV1 receptor plays a role in regulating emotional states and anxiety-like behavior and therefore some of the effects of anandamide might be mediated via this receptor [79, 80]. Finally, it cannot be ruled out that anandamide mediates some of its effects via a yet undiscovered mechanism.

In the small open field, we found a relatively small cannabis smoke-induced decrease in locomotor activity and a large decrease in rearing. It cannot completely be ruled out that some of these effects were partly mediated by stress associated with the cannabis smoke exposure. However, it should be noted that in previous studies we showed that exposure to tobacco smoke using a similar apparatus and for a long period of time (2–4 h) did not affect locomotor activity or operant responding for rewarding intracranial self-stimulation [34, 36]. Several studies reported that nose-only exposure to placebo cannabis smoke decreases locomotor activity [61, 62]. However, in these studies the mice were restrained during the exposure sessions, which contributed to the effects of placebo cannabis smoke on locomotor activity [61]. Other studies also reported that restraint stress decreases locomotor activity in rats [81, 82]. Furthermore, we found that CB_1_ receptor blockade completely prevented the cannabis smoke-induced decrease in rearing (Fig 4B). Therefore, based on our previous tobacco smoke exposure studies and the fact that CB_1_ receptor blockade prevented the effects of cannabis smoke on rearing, it is suggested that the effects of cannabis smoke on behavior were mainly due to CB_1_ receptor activation and not the stress of smoke exposure.

The large open field test and the elevated plus maze test are widely used to assess anxiety-like behavior in rodents. An increase in the amount of time spent or distance traveled in the center of a large open field without changes in locomotor activity is often interpreted as a decrease in anxiety-like behavior [68]. In the elevated plus maze test, an increase in the time on the open arms and an increase in the open / closed arm entry ratio also reflects a decrease in anxiety-like behavior [53]. In the present study, rats were tested in the elevated plus maze 48 h after cannabis smoke exposure and, on the following day, immediately after smoke exposure. During the first elevated plus maze test (48 h post-smoke), there were no differences between the air-control rats and cannabis rats in any of the parameters measured. Immediately after smoke exposure the cannabis rats spent more time exploring the closed arms and the center area but did not alter their exploration of the open arms. Anxiogenic effects could not be evaluated because of the very small amount of time that the control rats spent on the open arms. The rats were also tested in the large open field 48 h after smoke and, on the following day, immediately after smoke exposure. Cannabis smoke exposure increased the total time in the inner zone and decreased latency to enter the inner zone and the center zone. Although the post hoc comparisons did not reveal any significant effects, a close look at the data (S6 Table) reveals that in the cannabis rats the latency to enter the inner zone was decreased during the first and second session and the time in the inner zone was increased during the first and second session. The post hoc analyses showed that during the second session cannabis smoke exposure significantly decreased latency to enter the center of the open field. This pattern of results could suggest that exposure to cannabis smoke has anxiolytic-like effects. However, cannabis smoke also increased the total distance traveled and distance traveled in the outer zone. Therefore, it is most likely that the effects of cannabis smoke on anxiety-like parameters were due to a general increase in locomotor activity. This is supported by the results of the elevated plus maze test in which acute cannabis smoke exposure increased exploration of the closed arms but not the open arms. Thus, the present studies suggest that the cannabis smoke exposure conditions employed here do not decrease anxiety-like behavior.

Two cannabis smoke experiments (experiment 1 and experiment 3) were conducted and in both, body weights were recorded immediately before each smoke exposure session. In experiment 1 (8 weeks of exposure), cannabis smoke exposure did not affect body weight gain, but in experiment 3 (2 weeks of exposure) cannabis smoke exposure slightly attenuated weight gain. The discrepancy between these experiments might have been due to the fact that at the beginning of experiment 3 the body weights were lower than those at the beginning of experiment 1. Younger animals have a higher growth rate and therefore the smoke might have had a greater effect on body weight gain in experiment 3 [83]. Another difference between the two studies was the number of animals per group. In the first experiment there were 10 animals per group and in the third experiment there were 20 animals per group. Larger group sizes increase statistical power and the probability of detecting an effect of an experimental treatment [84]. The effect of cannabis smoke on body weight gain in rodents has not been thoroughly investigated. However, one study in mice reported that one week of cannabis smoke exposure did not affect weight gain but that 3 weeks of smoke exposure led to a small amount of weight loss [63]. These findings are in contrast to tobacco smoke exposure studies that consistently show that exposure to smoke significantly attenuates body weight gain in rodents [35, 36].

In conclusion, the data presented here indicate that exposure to cannabis smoke leads to clinically-relevant Δ9-THC levels and development of dependence as assessed by somatic withdrawal signs. It was also shown that acute cannabis smoke exposure induces a brief increase in locomotor activity followed by a prolonged decrease in locomotor activity and rearing. Furthermore, a high dose of anandamide decreases locomotor activity and rearing. Finally, blockade of CB_1_ receptors or prior exposure to cannabis smoke did not diminish the acute effects of a high dose of anandamide. This cannabis smoke exposure model should be useful for investigating the neurobiological mechanisms that mediate the effects of cannabis smoke on brain function.

## Supporting Information

S1 Fig Effect of cannabis smoke on body weight gain. Effect of 8 weeks (A) or 2 weeks (B) of cannabis smoke exposure on body weight gain. B: Asterisks (**p<0.01) indicate a lower body weight gain in the cannabis group than in the air-control group. N = 10 per group (A), N = 20 per group (B). Data expressed as means ± SEM.
(TIF)

S2 Fig locomotor activity and rearing in the small open field before smoke exposure. The rats were tested in the small open field five days before the onset of the smoke exposure sessions and horizontal (A) and vertical beam breaks (B) were assessed. There were no differences in horizontal and vertical beam breaks between the two groups before cannabis smoke exposure. N = 10 per group. Abbreviation: Air, air-control group. Data expressed as means ± SEM.
(TIF)

S3 Fig Cannabis smoke induces brief increase in locomotor activity in the small open field. The rats were tested in the small open field immediately after cannabis smoke exposure and the distance traveled was assessed. Asterisk (* p<0.05) indicates a significant difference from the air group. N = 10 per group. Abbreviation: Air, air-control group. Data expressed as means ± SEM.
(TIF)

S1 Table Effect of cannabis smoke on body weight gain. Asterisk (**p<0.01) indicate higher body weight than before onset of exposure sessions. Plus sign (+p<0.05) indicate lower body weight compared air-control rats at the end of the exposure period. Experiment 1, N = 10 / group; Experiment 3, N = 20 / group. Data are expressed as means ± SEM.
(DOC)

S2 Table Cannabis smoke and behavior in the small open field. Asterisk (*p<0.05, **p<0.01) indicate decreased horizontal or vertical beam breaks compared to the air-control group. N = 10 per group.
(DOC)

S3 Table Somatic withdrawal signs associated with precipitated cannabis withdrawal. Abdominal constrictions include gasps and writhes; facial fasciculations include cheek tremors, chews, and teeth chattering; shakes include head shakes and body shakes. Asterisks (*p<0.05, **p<0.01) indicate more withdrawal signs compared to the corresponding vehicle group. Plus signs (++p<0.01) indicate more somatic signs compared to the air-rimonabant group. N = 10 / group. Data are expressed as means ± SEM.
(DOC)

S4 Table Effect of rimonabant on the behavior of cannabis smoke exposed rats in the small open field. Baseline is 4 h after smoke exposure and before the systemic administration of rimonabant or vehicle. The rats were tested for an additional 45-min after the administration of rimonabant or vehicle.
(DOC)

S5 Table Cannabis smoke and behavior in the elevated plus maze. Rats were tested in the elevated plus maze 48 h after cannabis smoke exposure (Test 1) and the following day immediately after smoke exposure (Test 2). Plus signs (+p<0.05, ++p<0.01) indicate significant different from the same experimental group during test 1. Asterisks (*p<0.05) indicate significant different from air-control group during same test day.
(DOC)

S6 Table Cannabis smoke and behavior in the large open field. Rats were tested in the large open field 48 h after cannabis smoke exposure (Test 1) and the following day immediately after smoke exposure (Test 2). Plus signs (+p<0.05, ++p<0.01) indicate significant different from the same experimental group during test 1. Asterisks (*p<0.05, **p<0.01) indicate significant different from air-control group during same test day.
(DOC)

S7 Table Anandamide and behavior in the small open field. Asterisks (*p<0.05) indicate significant different from the vehicle (dose 0) group. N = 10 per group.
(DOC)

S8 Table Anandamide and behavior in the large open field. Asterisks (*p<0.05, **p<0.01) indicate significant different from the vehicle (dose 0) group. N = 10 per group.
(DOC)

S9 Table Effect of rimonabant on anandamide-induced behavioral changes in the small open field. Asterisks (**p<0.01) indicate significant different from the vehicle-vehicle group. N = 10–16 per group.
(DOC)

S10 Table Effect of cannabis smoke exposure on anandamide-induced behavioral changes in the small open field. Asterisk (*p<0.05) indicate significant different from the air-vehicle group. N = 10 per group.
(DOC)
