# Supplementary material for: Behavioral Characterization of the Effects of Cannabis Smoke and Anandamide in Rats
Source: PLoS One. 2016 Apr 11;11(4):e0153327. doi: 10.1371/journal.pone.0153327 (PMC4827836; doi:10.1371/journal.pone.0153327)
Supplement: S1 Table — Asterisk (**p<0.01) indicate higher body weight than before onset of exposure sessions. Plus sign (+p<0.05) indicate lower body weight compared air-control rats at the end of the exposure period. Experiment 1, N = 10 / group; Experiment 3, N = 20 / group. Data are expressed as means ± SEM. (DOC) [file pone.0153327.s004.doc]

**S1 Table.** Effect of cannabis smoke on body weight gain.

| Experiment | Air | | Cannabis | |
| --- | --- | --- | --- | --- |
|  | Baseline | End of exposure period | Baseline | End of exposure period |
| Experiment 1 (8 weeks) | 291.9 ± 4.8 | 469.4 ± 12.5** | 301.7 ± 3.6 | 495.5 ± 10.6** |
| Experiment 3 (2 weeks) | 217.4 ± 2.5 | 299.7 ± 2.9** | 215.4 ± 2.2 | 288.2 ± 2.6**+ |
