# Supplementary material for: Behavioral Characterization of the Effects of Cannabis Smoke and Anandamide in Rats
Source: PLoS One. 2016 Apr 11;11(4):e0153327. doi: 10.1371/journal.pone.0153327 (PMC4827836; doi:10.1371/journal.pone.0153327)
Supplement: S2 Table — Asterisk (*p<0.05, **p<0.01) indicate decreased horizontal or vertical beam breaks compared to the air-control group. N = 10 per group. (DOC) [file pone.0153327.s005.doc]

**S2 Table.** Cannabis smoke and behavior in the small open field.

| **Behavior** | | **Treatment** | |
| --- | --- | --- | --- |
|  |  | **Air** | **Cannabis** |
| Baseline before smoke exposure (30-min) | Horizontal beam breaks | 11919 ± 466 | 11454 ± 280 |
|  | Vertical beam breaks | 970 ± 73 | 1044 ± 59 |
| Immediately after smoke exposure (30-min) | Horizontal beam breaks | 10777 ± 678 | 8498 ± 445* |
|  | Vertical beam breaks | 1024 ± 83 | 636 ± 52** |
