# Supplementary material for: Behavioral Characterization of the Effects of Cannabis Smoke and Anandamide in Rats
Source: PLoS One. 2016 Apr 11;11(4):e0153327. doi: 10.1371/journal.pone.0153327 (PMC4827836; doi:10.1371/journal.pone.0153327)
Supplement: S3 Table — Abdominal constrictions include gasps and writhes; facial fasciculations include cheek tremors, chews, and teeth chattering; shakes include head shakes and body shakes. Asterisks (*p<0.05, **p<0.01) indicate more withdrawal signs compared to the corresponding vehicle group. Plus signs (++p<0.01) indicate more somatic signs compared to the air-rimonabant group. N = 10 / group. Data are expressed as means ± SEM. (DOC) [file pone.0153327.s006.doc]

**S3 Table.** Somatic withdrawal signs associated with precipitated cannabis withdrawal.

| **Behavior** | **Air** | | **Cannabis** | |
| --- | --- | --- | --- | --- |
|  | **Vehicle** | **Rimonabant** | **Vehicle** | **Rimonabant** |
| Abdominal constrictions | 0.1± 0.1 | 0.2 ± 0.2 | 0.4 ± 0.2 | 1.2 ± 0.3 |
| Digging | 0 | 0 | 0.1 ± 0.1 | 0.3 ± 0.2 |
| Eye blinks | 3.9 ± 0.5 | 2.5 ± 0.4 | 4.7 ± 0.7 | 7.6 ± 1.2++* |
| Facial fasciculations | 0 | 0.1 ± 0.1 | 0.5 ± 0.5 | 0.7 ± 0.3 |
| Forepaw fluttering | 0.3± 0.1 | 0.6 ± 0.3 | 0.1 ± 0.1 | 1.8 ± 0.8* |
| Genital licks | 0 | 0 | 0 | 0.5 ± 0.2 |
| Grooming | 1.7± 0.3 | 4.1 ± 0.5* | 1.7 ± 0.5 | 8.8 ± 1.2++** |
| Ptosis | 0 | 0.4 ± 0.1 | 0 | 2.1 ± 0.8* |
| Shakes | 0.2± 0.2 | 2.5 ± 0.8* | 0.2 ± 0.1 | 3.1 ± 0.8* |
| Yawns | 0 | 0 | 0.2 ± 0.2 | 0.4 ± 0.2 |
