# Supplementary material for: Behavioral Characterization of the Effects of Cannabis Smoke and Anandamide in Rats
Source: PLoS One. 2016 Apr 11;11(4):e0153327. doi: 10.1371/journal.pone.0153327 (PMC4827836; doi:10.1371/journal.pone.0153327)
Supplement: S4 Table — Baseline is 4 h after smoke exposure and before the systemic administration of rimonabant or vehicle. The rats were tested for an additional 45-min after the administration of rimonabant or vehicle. (DOC) [file pone.0153327.s007.doc]

**S4 Table.** Effect of rimonabant on the behavior of cannabis smoke exposed rats in the small open field.

| **Behavior** | | **Air** | | **Cannabis** | |
| --- | --- | --- | --- | --- | --- |
|  |  | **Vehicle** | **Rimonabant** | **Vehicle** | **Rimonabant** |
| Pre-drug injections (baseline, 30 min) | Horizontal beam breaks | 9774 ± 355 | 10069 ± 422 | 8669 ± 298 | 8907 ± 520 |
|  | Vertical beam breaks | 971 ± 72 | 1000 ± 70 | 748 ± 59 | 819 ± 70 |
| Post-drug injections (45 min) | Horizontal beam breaks | 8219 ± 623 | 10013 ± 535 | 6798 ± 411 | 8808 ± 655 |
|  | Vertical beam breaks | 737 ± 84 | 778 ± 82 | 474 ± 45 | 766 ± 77 |
