# Supplementary material for: Behavioral Characterization of the Effects of Cannabis Smoke and Anandamide in Rats
Source: PLoS One. 2016 Apr 11;11(4):e0153327. doi: 10.1371/journal.pone.0153327 (PMC4827836; doi:10.1371/journal.pone.0153327)
Supplement: S5 Table — Rats were tested in the elevated plus maze 48 h after cannabis smoke exposure (Test 1) and the following day immediately after smoke exposure (Test 2). Plus signs (+p<0.05, ++p<0.01) indicate significant different from the same experimental group during test 1. Asterisks (*p<0.05) indicate significant different from air-control group during same test day. (DOC) [file pone.0153327.s008.doc]

**S5 Table.** Cannabis smoke and behavior in the elevated plus maze.

| **Behavior** | | **Test 1** | | **Test 2** | |
| --- | --- | --- | --- | --- | --- |
|  |  | **Air** | **Cannabis** | **Air** | **Cannabis** |
| All zones (open and closed arms, and center) | Total distance traveled (cm) | 1356 ± 70 | 1411 ± 49 | 1494 ± 67+ | 1779 ± 70++* |
|  | Time moving (s) | 176 ± 8 | 185 ± 5 | 166 ± 5 | 185 ± 5* |
| Distance moved in specific zone (s) | Closed arms | 1121 ± 64 | 1130 ± 36 | 1178 ± 52 | 1374 ± 50++* |
|  | Open arms | 27 ± 11 | 55 ± 16 | 37 ± 15 | 47 ± 31 |
|  | Center | 207 ± 14 | 226 ± 28 | 279 ± 25+ | 358 ± 25++ |
| Duration in specific zone (s) | Closed arms | 225 ± 7 | 218 ± 11 | 217 ± 10 | 202 ± 9 |
|  | Open arms | 10 ± 4 | 17 ± 5 | 10 ± 4 | 11 ± 5 |
|  | Center | 65 ± 4 | 66 ± 9 | 73 ± 7 | 87 ± 7+ |
|  | Open / total* % | 4 ± 2 | 7 ± 2 | 5 ± 2 | 5 ± 2 |
| Frequency of arm entries | Closed arms | 17 ± 2 | 16 ± 1 | 20 ± 2 | 22 ± 2+ |
|  | Open arms | 4 ± 1 | 5 ± 1 | 2 ± 1+ | 4 ± 1 |
|  | Open / total % | 22 ± 6 | 22 ± 3 | 10 ± 3+ | 15 ± 2 |

* Total is time on open and closed arms.
