# Supplementary material for: Behavioral Characterization of the Effects of Cannabis Smoke and Anandamide in Rats
Source: PLoS One. 2016 Apr 11;11(4):e0153327. doi: 10.1371/journal.pone.0153327 (PMC4827836; doi:10.1371/journal.pone.0153327)
Supplement: S6 Table — Rats were tested in the large open field 48 h after cannabis smoke exposure (Test 1) and the following day immediately after smoke exposure (Test 2). Plus signs (+p<0.05, ++p<0.01) indicate significant different from the same experimental group during test 1. Asterisks (*p<0.05, **p<0.01) indicate significant different from air-control group during same test day. (DOC) [file pone.0153327.s009.doc]

**S6 Table.** Cannabis smoke and behavior in the large open field.

| **Behavior** | | **Test 1** | | **Test 2** | |
| --- | --- | --- | --- | --- | --- |
|  |  | **Air** | **Cannabis** | **Air** | **Cannabis** |
| All zones (border, middle, and center) | Total distance traveled (cm) | 3925 ± 192 | 4070 ± 188 | 3104 ± 232+ | 4087 ± 183** |
|  | Time moving (s) | 242 ± 6 | 245 ± 4 | 196 ± 8++ | 232 ± 7** |
| Latency to enter zone (s) | Inner | 59 ± 11 | 26 ± 7 | 97 ± 28 | 37 ± 7 |
|  | Center | 116 ± 18 | 148 ± 28 | 178 ± 33 | 78 ± 22* |
| Duration in zone (s) | Outer | 279 ± 3 | 266 ± 6 | 285 ± 4 | 271 ± 6 |
|  | Inner | 16 ± 2 | 27 ± 5 | 12 ± 3 | 22 ± 5 |
|  | Center | 5 ± 1 | 7 ± 2 | 5 ± 1 | 8 ± 2 |
| Distance traveled in zone (cm) | Outer | 3488 ± 161 | 3394 ± 98 | 2771 ± 186+ | 3560 ± 157** |
|  | Inner | 348 ± 56 | 534 ± 99 | 254 ± 55 | 392 ± 71 |
|  | Center | 89 ± 17 | 141 ± 35 | 78 ± 22 | 136 ± 27 |
