# Supplementary material for: Behavioral Characterization of the Effects of Cannabis Smoke and Anandamide in Rats
Source: PLoS One. 2016 Apr 11;11(4):e0153327. doi: 10.1371/journal.pone.0153327 (PMC4827836; doi:10.1371/journal.pone.0153327)
Supplement: S7 Table — Asterisks (*p<0.05) indicate significant different from the vehicle (dose 0) group. N = 10 per group. (DOC) [file pone.0153327.s010.doc]

**S7 Table.** Anandamide and behavior in the small open field.

| **Behavior** | **Dose of anandamide (mg/kg)** | | | | |
| --- | --- | --- | --- | --- | --- |
|  | **0** | **0.01** | **0.1** | **1** | **10** |
| Horizontal beam breaks | 14489 ± 1208 | 17248 ± 776 | 14926 ± 1204 | 17209 ± 843 | 11395 ± 692 |
| Vertical beam breaks | 1100 ± 71 | 1238 ± 66 | 1226 ± 91 | 1400 ± 67* | 785 ± 79* |
