# Supplementary material for: Behavioral Characterization of the Effects of Cannabis Smoke and Anandamide in Rats
Source: PLoS One. 2016 Apr 11;11(4):e0153327. doi: 10.1371/journal.pone.0153327 (PMC4827836; doi:10.1371/journal.pone.0153327)
Supplement: S8 Table — Asterisks (*p<0.05, **p<0.01) indicate significant different from the vehicle (dose 0) group. N = 10 per group. (DOC) [file pone.0153327.s011.doc]

**S8 Table.** Anandamide and behavior in the large open field.

| **Behavior** | | **Dose of anandamide (mg/kg)** | | | | |
| --- | --- | --- | --- | --- | --- | --- |
|  |  | **0** | **0.01** | **0.1** | **1** | **10** |
| All zones (border, middle, and center) | Total distance traveled (cm) | 6531 ± 436 | 6453 ± 389 | 6628 ± 446 | 5410 ± 558 | 2480 ± 516** |
|  | Time moving (s) | 437 ± 18 | 424 ± 16 | 437 ± 15 | 444 ± 22 | 228 ± 31** |
| Latency to enter zone (s) | Middle | 70 ± 31 | 35 ± 10 | 46 ± 16 | 105 ± 53 | 325 ± 84** |
|  | Center | 153 ± 37 | 170 ± 50 | 151 ± 45 | 212 ± 70 | 480 ± 71** |
| Duration in zone (s) | Border | 547 ± 10 | 561 ± 7 | 552 ± 7 | 541 ± 9 | 588 ± 5** |
|  | Middle | 37 ± 6 | 30 ± 5 | 36 ± 5 | 41 ± 6 | 9 ± 4** |
|  | Center | 16 ± 4 | 9 ± 2 | 12 ± 3 | 19 ± 4 | 3 ± 2* |
| Distance traveled in zone (cm) | Border | 5407 ± 273 | 5614 ± 288 | 5793 ± 349 | 5515 ± 394 | 2275 ± 409** |
|  | Middle | 850 ± 151 | 685 ± 122 | 651 ± 99 | 808 ± 138 | 152 ± 80** |
|  | Center | 275 ± 59 | 155 ± 31 | 185 ± 45 | 278 ± 58 | 53 ± 33* |
