# Supplementary material for: Behavioral Characterization of the Effects of Cannabis Smoke and Anandamide in Rats
Source: PLoS One. 2016 Apr 11;11(4):e0153327. doi: 10.1371/journal.pone.0153327 (PMC4827836; doi:10.1371/journal.pone.0153327)
Supplement: S9 Table — Asterisks (**p<0.01) indicate significant different from the vehicle-vehicle group. N = 10–16 per group. (DOC) [file pone.0153327.s012.doc]

**S9 Table.** Effect of rimonabant on anandamide-induced behavioral changes in the small open field.

| **Behavior** | **Vehicle** | | **Rimonabant** | |
| --- | --- | --- | --- | --- |
|  | **Vehicle** | **Anandamide** | **Vehicle** | **Anandamide** |
| Horizontal beam breaks | 6182 ± 216 | 4332 ± 277** | 5458 ± 311 | 4058 ± 348** |
| Vertical beam breaks | 571 ± 34 | 236 ± 48** | 554 ± 60 | 291 ± 55** |
