# Supplementary material for: Behavioral Characterization of the Effects of Cannabis Smoke and Anandamide in Rats
Source: PLoS One. 2016 Apr 11;11(4):e0153327. doi: 10.1371/journal.pone.0153327 (PMC4827836; doi:10.1371/journal.pone.0153327)
Supplement: S10 Table — Asterisk (*p<0.05) indicate significant different from the air-vehicle group. N = 10 per group. (DOC) [file pone.0153327.s013.doc]

**S10 Table.** Effect of cannabis smoke exposure on anandamide-induced behavioral changes in the small open field.

| **Behavior** | **Air** | | **Cannabis** | |
| --- | --- | --- | --- | --- |
|  | **Vehicle** | **Anandamide** | **Vehicle** | **Anandamide** |
| Horizontal beam breaks | 14061 ± 877 | 11738 ± 809 | 10996 ± 901* | 12355 ± 549 |
| Vertical beam breaks | 1007 ± 104 | 854 ± 110 | 831 ± 104 | 877 ± 72 |
